# Laparoscopic appendectomy for acute appendicitis in patients with COVID-19 confirmation: A case report

**DOI:** 10.1016/j.ijscr.2022.107740

**Published:** 2022-10-11

**Authors:** Toshiyuki Suzuki, Akiyo Matsumoto, Takahiko Akao, Seiji Kobayashi, Hiroshi Matsumoto

**Affiliations:** Department of Surgery, Hanyu General Hospital, Hanyushi, Saitama 348-8505, Japan

**Keywords:** COVID-19, coronavirus disease 2019, CT, computed tomography, PPE, personal protective equipment, SARS-Cov-2, severe acute respiratory syndrome coronavirus 2, NOM, non-operative management, RT-PCR, real-time polymerase chain reaction, COVID-19, SARS CoV-2, Emergency surgery, Laparoscopic appendectomy, Case report

## Abstract

**Introduction:**

Strategies to postpone elective surgeries were proposed to maintain the hospital capacity to cater for coronavirus disease 2019 (COVID-19) and emergency non-COVID cases. Non-operative management (NOM) was recommended when possible during the COVID-19 era. However, the optimal approach to acute appendicitis (AA) in patients with COVID-19 remains controversial.

**Presentation of case:**

A 25-year-old man who tested positive for severe acute respiratory syndrome coronavirus 2 (SARS-Cov-2) was referred to our institution with a diagnosis of AA with appendicolith. Chest computed tomography did not detect evidence of pneumonia. Laparoscopic appendectomy was performed after strict infection prevention measures were taken. The postoperative course was uneventful. No respiratory symptoms such as cough or sputum production occurred postoperatively. No signs of infection in medical staff or spread in the operating room and infectious disease ward were observed.

**Discussion:**

The treatment policy should fully consider the risk of COVID-19 infection to medical staff and the risk of aggravation in patients who tested positive for SARS-Cov-2. Surgery was chosen over NOM for AA with appendicolith because the presence of appendicolith was thought to indicate a high probability of treatment failure in NOM and possible perforation; thus, case more difficult measures were required for SARS-Cov-2-positive cases.

**Conclusion:**

Careful assessment of the patient's condition and consideration of the treatment method is important, rather than choosing NOM over operative management based solely on SARS-Cov-2-positive status. Laparoscopic appendectomy with adequate infection control measures can be safely performed in SARS-Cov-2-positive cases.

## Highlights

• Treatment policy should consider COVID-19 risk to medical staff and aggravation.
• Laparoscopic appendectomy with adequate infection control can be safe in COVID-19.
• Enhanced PPE is needed to prevent infections in the operating room and wards.

## 1 Introduction

Coronavirus disease-2019 (COVID-19) was confirmed in December 2019, causing an unprecedented pandemic worldwide. As a result, surgical care has undergone major changes. In addition, various guidelines were made on how to perform emergency surgery during the COVID-19 era [1]. Strategies to postpone elective surgeries were also proposed to maintain the hospital capacity to cater for severe acute respiratory syndrome coronavirus 2 (SARS-Cov-2)-positive and emergency SARS-Cov-2-negative cases, and non-operative management (NOM) was recommended when possible [2], [3]. However, early surgery may be effective in some cases, such as this case in which COVID-19 was confirmed.

Herein, we report an adult case of laparoscopic appendectomy performed on a patient with COVID-19. This report aimed to provide information on the safety and usefulness of laparoscopic appendectomy patients with COVID-19, even when adequate infection control is in place. This case report has been reported in line with the SCARE 2020 criteria [4].

## 2 Presentation of case

A 25-year-old man with a diagnosis of acute appendicitis (AA) with appendicolith was referred to our institution for surgery. The patient had a fever (body temperature (BT) of 38 °C) 13 days prior to referral and received home treatment (quarantined for 10 days) after testing positive for SARS-Cov-2 by real-time polymerase chain reaction (RT-PCR) analysis 12 days before the transfer. The patient had no complaints including respiratory symptoms at the time of home treatment. Lower abdominal pain began the day before referral, and he was diagnosed with AA on a non-contrast abdominal computed tomography (CT) and tested positive for SARS-Cov-2 antigen.

**Fig. 1:**
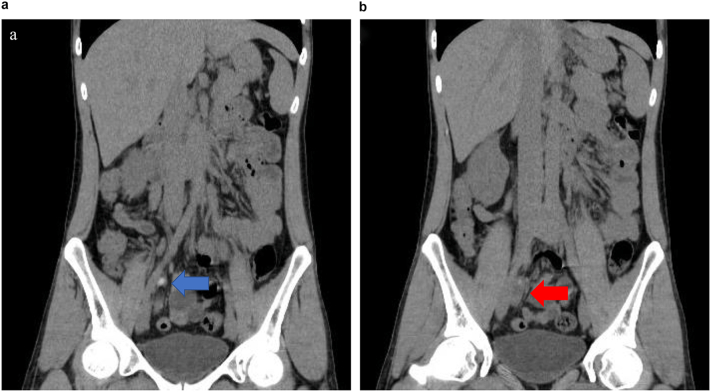
Computed tomography findings. (A) Appendicolith (blue arrow) was found in the appendix. (B) A swollen appendix was noted (red arrow). (For interpretation of the references to color in this figure legend, the reader is referred to the web version of this article.) (For interpretation of the references to color in this figure legend, the reader is referred to the web version of this article.)

**Fig. 2:**
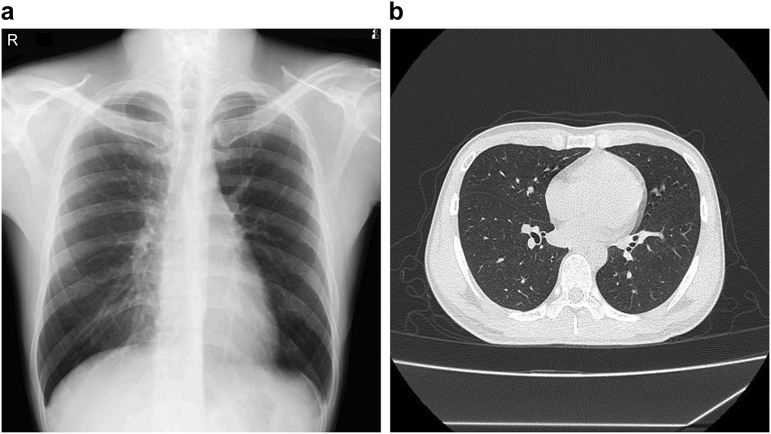
Chest X-ray and chest CT findings (A) Chest X-ray showed no abnormal shadows in the lung field. (B) Chest CT showed no pneumonia CT, computed tomography.

Lower abdominal pain and tenderness were noted, but there was no rigidity, rebound tenderness, or any respiratory symptoms such as coughing or sputum production. His vital signs were as follows: blood pressure of 116/72 mmHg, pulse rate of 79 beats/min, respiratory rate of 12 times/min, BT of 37.3 °C, and 99 % oxygen saturation on room air. Laboratory tests revealed a white blood cell count of 12,290 cells/μL and C-reactive protein of 1.95 mg/dL. CT revealed an enlarged appendix with appendicolith (Fig. 1). No evidence of pneumonia was found on chest X-ray imaging and CT (Fig. 2). RT-PCR analysis performed in our institution confirmed SARS-Cov-2 infection.

Based on the above, our diagnosis was SARS-Cov-2-positive AA and selected emergency surgery. NOM was not considered because of appendicitis accompanied by appendicolith [5]. In addition, positivity to SARS-Cov-2 was thought to make emergency surgical procedures more difficult if NOM failed. Moreover, because of the holidays, there were no scheduled surgeries, and the operating room was available. Laparoscopic appendectomy was less invasive and could be completed in a short period, which was considered beneficial to the outcomes without the risk of further spread of infection. The usefulness of laparoscopic appendectomy was also reported [6]. Thus, laparoscopic appendectomy was performed by a gastrointestinal surgeon at a district general hospital.

**Fig. 3:**
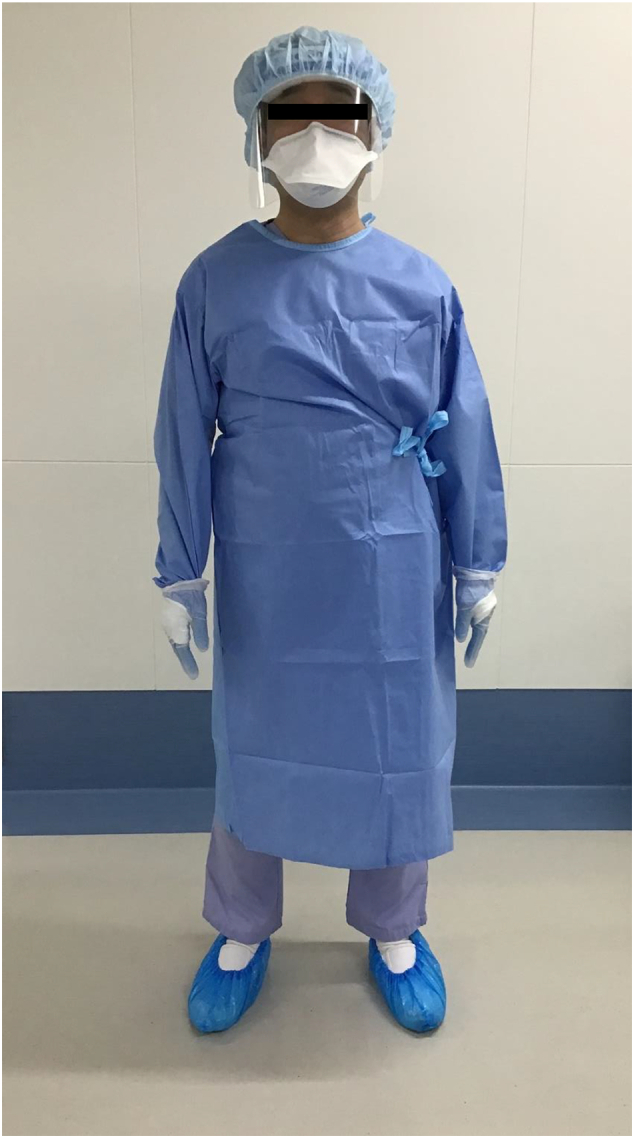
Enhanced personal protective equipment.

All members of the surgical team, including the anesthesiologist, wore enhanced personal protective equipment. The enhanced PPE included a surgical gown, head cover, face shield, two pairs of surgical gloves, shoe covers, and an N95 mask [7] (Fig. 3). The anesthesiologist induced general anesthesia was through endotracheal intubation using a video laryngoscope.

A 12-mm port was used for the navel, and a 5-mm port was used for the upper pubic area and the left paramedian plane. Pneumoperitoneum was induced up to an abdominal pressure of 10 mmHg at a CO₂ flow rate of 40 L/min. A closed circuit smoke exhaust system was not used. Ultrasonic coagulation and incision device were not used because they produce large surgical smoke particles at relatively low temperatures, which are reportedly likely to contain viable viruses [8]. Peeling operation was performed by energizing a general peeling forceps, and soft coagulation using an IO advanced electrode was mainly used for hemostasis operation. The appendix was also mildly adherent to the surrounding tissue. The appendix was swollen, but no perforation was found. The mesentery of the appendix was divided using endoclips, and the base of the appendix was ligated with chromic ENDOLOOPS®. The appendix was placed in a plastic bag and removed. Fecaliths were found inside the appendix.

The operation lasted 79 min, anesthesia was applied for 115 min, and the operating room retention time was 135 min. Anesthesia awakening and extubation were performed in the operating room, and the patients was then moved to the infectious disease ward.

The postoperative course was unremarkable, and the patient was discharged on postoperative day 7. No respiratory symptoms such as cough or sputum production occurred postoperatively. The pathological finding was phlegmonous appendicitis. No infection was found in all participating members of the surgery team. No signs of infection in medical staff or spread in the operating room and infectious disease ward were observed. Nearly all medical staff were vaccinated.

## 3 Discussion

Recommendations regarding surgical indications for SARS-Cov-2-positive or suspected cases include deferring surgery unless cases are potentially fatal or severe [1], [2], [3]. However, there are cases, such as this SARS-Cov-2-positive case, where emergency surgery is useful. Careful assessment of the patient's condition and consideration of the treatment method are essential, rather than choosing NOM over surgical treatment based solely on the SARS-Cov-2-positive state.

If a cluster occurs at a core hospital that is responsible for regional medical care, such as our hospital, regional medical care may collapse. Therefore, full consideration of the COVID-19 infection risk of the medical staff and wards is necessary before deciding on treatment strategies.

Previously, we performed surgery for SARS-Cov-2-positive sigmoid volvulus, perforated appendicitis, and pregnant cases. In addition, an operating room nurse worked in the infectious disease ward on a rotation basis for 1 month since the start of the COVID-19 pandemic. Medical staff also studied infection prevention measures related to COVID-19. Based on these experiences, SARS-Cov-2-positive emergency laparoscopic appendectomy could have been performed more safely.

Surgery was chosen over NOM for AA with appendicolith [5]. The presence of appendicolith has been identified as an independent prognostic risk factor for treatment failure in NOM in uncomplicated AA [9]. Moreover, AA with appendicolith is associated with increased perforation risk [10]. If NOM were chosen in this case, treatment was likely to fail and resulted in perforation, necessitating emergency surgery, and a more difficult response given the presence of COVID-19.

Considering the risk of spreading COVID-19, there remains controversies over whether laparotomy or laparoscopy is better. Surgical smoke may contain chemicals, bacteria, viruses, etc., and medical workers are at risk of health hazards [11]. Surgical smoke may also contain SARS-CoV-2. Laparoscopic surgery has been reported to increase the risk of infection among surgical staff because it generates higher surgical smoke concentration than open surgery [12]. However, laparoscopic surgery may have a lower risk of infection if adequate infection control measures are taken [11], [13]. Based on the above, we chose laparoscopic surgery.

Surgery for SARS-Cov-2-positive cases involves not only the risk of spreading infections to medical staff and wards but also the risk of aggravation of patients' condition. In addition, postoperative pneumonia and cardiovascular complications have been reported in SARS-Cov-2-positive cases, of which some have become severe and patients even died [14], [15]. Therefore, as recommended by surgical societies worldwide, it is important to postpone surgery or consider alternative medicine for patients who can wait. However, even in SARS-Cov-2-positive cases such as this case, laparoscopic appendectomy can be safely performed by taking measures against the medical situation and individual case risks.

## 4 Conclusion

Careful assessment of the patient's condition and consideration of the treatment method are important, rather than choosing NOM over operative management based solely on the SARS-Cov-2-positive state. Even in SARS-Cov-2-positive cases such as this case, laparoscopic appendectomy with sufficient infection control measures can be safely performed.

## Consent

Written informed consent was obtained from the patient for publication of this case report and accompanying images. A copy of the written consent is available for review by the Editor-in-Chief of this journal on request.

## Provenance and peer review

Not commissioned, externally peer-reviewed.

## Sources of funding

None.

## Research registration

None.

## Ethical approval

IRB/Ethics Committee of Hanyu General Hospital ruled that approval was not required for this study.

## Guarantor

Toshiyuki Suzuki

## CRediT authorship contribution statement

**Toshiyuki Suzuki:** Conceptualization, Methodology, Software, Validation, Formal analysis, Investigation, Resources, Writing – original draft, Visualization, Project administration, Funding acquisition. **Akiyo Matsumoto:** Data curation, Supervision. **Takahiko Akao:** Data curation. **Seiji Kobayashi:** Data curation. **Hiroshi Matsumoto:** Writing – review & editing.

## Declaration of competing interest

None.
